# The prevalence of the metabolic syndrome in Portugal: the PORMETS study

**DOI:** 10.1186/s12889-017-4471-9

**Published:** 2017-06-08

**Authors:** Luís Raposo, Milton Severo, Henrique Barros, Ana Cristina Santos

**Affiliations:** 1 Insulin Resistance Study Group of the Portuguese Society of Endocrinology, Diabetes and Metabolism, Lisboa, Portugal; 2 EPIUnit - Instituto de Saúde Pública, Universidade do Porto, Rua das Taipas, n° 135, 4050-600 Porto, Portugal; 3 Departamento de Ciências da Saúde Pública e Forenses e Educação Médica, Faculdade de Medicina, Universidade do Porto, Porto, Portugal

**Keywords:** Metabolic syndrome, Prevalence, Portugal, Rural, Regional, Socio-economic

## Abstract

**Background:**

The PORMETS study was designed to estimate the prevalence of metabolic syndrome and its determinants in the overall and administrative regions of the Portuguese mainland.

**Methods:**

A cross-sectional study of a representative sample of non-institutionalized Portuguese adults selected from primary health care centres lists including 1695 men and 2309 women was conducted from February 2007 to July 2009. A structured questionnaire was administered, collecting information on personal medical history and socio-demographic and behavioural characteristics. Anthropometrics, blood pressure, and venous blood samples were obtained. Metabolic syndrome was defined according to three operational definitions. The prevalence ratios and their respective 95% confidence intervals were calculated using binomial generalized linear regression, with the log link function.

**Results:**

The prevalence rates of metabolic syndrome in this sample of Portuguese adults were 36.5%, 49.6%, and 43.1%, using the Adult Treatment Panel III, International Diabetes Federation and Joint Interim Statement definitions, respectively. The most prevalent feature of metabolic syndrome in this sample was high blood pressure (64.3%) and the lowest was high fasting glucose (24.9%). After adjustment for age and gender, significant differences were observed for the 18 districts of the Portugal mainland. Additionally, metabolic syndrome was significantly more frequent in non-urban areas than in urban ones (*p* = 0.001). The prevalence of metabolic syndrome was significantly higher in women (p˂0.001) and older participants (p˂0.001), as well as in those who reported being housewives (*p* = 0.010), retired (*p* = 0.046) or unemployed (*p* = 0.024).

**Conclusions:**

This study showed that metabolic syndrome is highly prevalent in the Portuguese adult population. Regional differences in the prevalence of this syndrome were observed, and this condition was more common in non-urban areas and less favoured socio-economic categories.

## Background

Metabolic syndrome (MetS) is a cluster of interrelated risk factors for cardiovascular disease (CVD) [1] and diabetes [2]. These factors include dysglycaemia, elevated blood pressure, elevated triglyceride levels, low high-density lipoprotein cholesterol (HDL-C) levels, and central obesity. Different organizations have proposed several diagnostic criteria [3–6]. However, finally, a consensus was established by the International Diabetes Federation (IDF), National Heart, Lung, and Blood Institute, American Heart Association and other international societies [7].

MetS is a common condition and its worldwide prevalence is rising [8], which can be related to the increase in obesity rates and sedentary lifestyles, as well as regional prevalence of hypertension and diabetes. The prevalence of overweight and obesity in Portugal is high [9] and is increasing [10]. Overall, individuals with normal weight (Wt) represent less than 50% of the adult population [10]. Recent studies have also shown a high prevalence of hypertension and type 2 diabetes in the Portuguese population (42.2% and 11.7%, respectively) [11, 12].

Previous national studies have shown the prevalence of MetS, ranging from 42% to 66%, according to the IDF criteria [13–15]. However, none of them approached district and urban versus non-urban differences.

The Portuguese Metabolic Syndrome (PORMETS) study, was designed to assess the prevalence of MetS and its determinants in the overall and administrative regions of the Portuguese mainland.

## Methods

PORMETS is a national cross-sectional study. A sample of adults registered at primary health care centres throughout mainland Portugal was selected. In each of the eighteen Portuguese mainland administrative regions (districts), two health care centres were included, one in the district’s capital and the other representative of a non-urban area. Apart from the district of Setubal that only included one centre, all the others included two, for a total of 35 health care centres. At each centre, the participants were randomly selected from the general practitioner’s patient lists, and 120 participants were evaluated, with an inclusion criterion of being aged 18 or older. A total of 4105 participants was evaluated, and information was collected from February 2007 to July 2009. Ten participants were excluded from the data analysis because they were pregnant at the time of the interview, resulting in 4095 remaining participants. After excluding participants who had missing information on the MetS features, the final analysis included 4004 participants, 2309 women and 1695 men.

All of the Portuguese Regional Health Administrations, the Ethics Committee of the São João Hospital E.P.E. and the Portuguese Data Protection Authority approved PORMETS. The Clinical Director of each health care centre also provided authorization and all participants provided written informed consent.

Anthropometrics measures were recorded, namely Wt, height, and waist and hip circumferences. Body Wt was measured to the nearest 0.1 kg using a digital scale, and height was measured to the nearest centimetre in the standing position using a wall stadiometer. Body mass index (BMI) was calculated as the Wt in kilogrammes divided by the square height in metres. The waist circumference (WC) was measured midway between the lower limit of the rib cage and the iliac crest and the hip circumference (HC) was measured as the maximum circumference of the buttocks. Blood pressure was measured on a single occasion using a standard mercury sphygmomanometer with the cuff on the upper right arm after a 10-min rest. Two blood pressure readings were taken, and the mean of the two readings was calculated. If the difference between the two measures was larger than 5 mmHg for systolic or diastolic blood pressure, a third measurement was acquired and the mean of the two closest values was registered.

Fasting venous blood glucose, total cholesterol and triglycerides levels were determined using automatic standard routine enzymatic methods. HDL-C was determined after the precipitation of apolipoprotein B-containing lipoproteins. All participants with triglycerides levels below 400 mg/dl had their LDL cholesterol level computed. This value was estimated by subtracting the HDL- C value plus 20% of the triglycerides from the total cholesterol. High sensitivity C-reactive protein (hs-CRP) levels were determined using particle-enhanced immunonephelometry. Insulin was measured using the ^125^I–labelled insulin radioimmunoassay method, and insulin resistance was estimated according to the homeostatic model assessment (HOMA), as the product of fasting glucose (mmol/L) and insulin (μUI/mL) divided by a constant 22.5.

A trained nurse administered a structured questionnaire with only closed ended questions; information was collected on personal medical history and socio-demographic and behavioural characteristics. A participant was considered a current smoker if he/she smoked daily or occasionally, a former smoker was considered a participant who had stopped smoking for at least six months, and a never smoker was considered a participant who had never smoked [16]. Regarding alcohol intake, the participants were categorized as occasional drinkers if he/she had less than a drink per day, daily drinker if he/she consumed, at least one drink per day, and a non-drinker if he/she did not consume any alcoholic beverages. Regular physical exercise was considered when the participant was engaged in some leisure-time physical activity performed on a repeated basis, spending at least 30 min a week.

Portuguese regions were classified as North, Center, Lisbon, Alentejo and Algarve according to the NUTS2 level statistical regions of the European Union.

Three operational definitions of MetS were used: the Adult Treatment Panel III (ATP III) [4], IDF [5], and HARM2009 [7]. MetS was considered present by ATP III if at least three (any) of the following characteristics were present: fasting glucose ≥110 mg/dL; blood pressure ≥ 130/85 mmHg; triglycerides ≥150 mg/dL; HDL-C < 40 mg/dL in women and <30 mg/dL in men; WC > 88 cm in women and >102 cm in men. Participants who reported the use of antihypertensive or antidiabetic therapy were also considered as having the corresponding MetS feature by the ATP III classification. The considered IDF definition was WC ≥ 80 cm in women and ≥94 cm in men and the presence of at least two of the following characteristics: fasting glucose ≥100 mg/dL or previously diagnosed type 2 diabetes; blood pressure ≥ 130/85 mmHg or antihypertensive medication; triglycerides ≥150 mg/dL or current treatment for this lipid abnormality; HDL-C < 40 mg/dL in women and <30 mg/dL in men or current treatment for this lipid abnormality. Finally, the HARM2009 defined MetS as the presence of at least three (any) of the following characteristics: fasting glucose ≥100 mg/dL or antidiabetic treatment; blood pressure ≥ 130/85 mmHg or antihypertensive medication; triglycerides ≥150 mg/dL or specific treatment for this lipid abnormality; HDL-C < 40 mg/dL in women and <30 mg/dL in men or specific treatment for this lipid abnormality; WC ≥ 88 cm in women and ≥102 cm in men (“European” cut off points).

### Statistical analysis

The data are described as the mean values and standard deviation (SD) or as median values and corresponding 25th and 75th percentiles for non-normally distributed variables. Counts and proportions were reported for categorical variables. Proportions were compared using the chi-square test or *Fisher’s* exact test, whenever appropriate. Student’s t test or Mann-Whitney *U*-test was used to compare continuous variables.

The prevalence of MetS and its individual components was age and sex adjusted, using binomial generalized linear regression, with the log link function.

The prevalence and prevalence ratio (PR) of MetS and their respective 95% confidence intervals (95%CI) were estimated for districts and NUT II by binomial generalized linear regression, with the log link function. Each district and NUT II region was compared with the overall effect, obtained as the pooled geometric mean prevalence of all districts, and the PR and respective 95%CI were calculated using the deviation contrast method [17].

To estimate the magnitude of association between MetS and demographic, socio-economic analytical and lifestyle characteristics, the PR and 95%CI were also computed using binomial generalized linear regression, with the log link function. The *p*-values were obtained using the Wald test from the respective generalized linear regressions.

Statistical analysis was performed using SPSS version 21 ® and R version 8.0 software (R Foundation, Vienna, Austria).

## Results

This study included 4004 individuals, 2309 women and 1695 men with a mean age of 53.2 (SD = 16.3) years. The mean age was 54.1 (SD = 16.3) years in men and 52.6 (SD = 16.3) years in women (*p* = 0.004). The mean (SD) levels of -glucose, triglycerides and HDL-C in women and men were, respectively, 88.7 (25.4) / 115.1 (58.5) / 50.9 (11.8) and 96.5 (30.5) / 135.1 (85.0) / 43.8 (12.3) mg/dL. The mean (SD) values of WC were 90.9 (12.5) cm in women and 97.0 (11.4) cm in men. Systolic and diastolic blood pressure values (mean and SD) were, respectively, 129.1 (22.2) / 77.2 (12.1) mmHg in women and 136.0 (21.8) / 79.8 (12.1) mmHg in men. Differences between sexes were significant (*p* < 0.001) for all.

The prevalence rates of MetS in this sample of Portuguese adults adjusted for gender and age were 36.5%, 49.6% and 43.1% (crude prevalence rates of 32.7%, 45.9% and 40.0%) using the ATPIII, IDF and HARM2009 definitions respectively (Table 1). The most prevalent feature of MetS in this sample was “high blood pressure” (64.3%) and the lowest was “high fasting glucose” (24.9%). Most participants with MetS had 3 features (23.3%). A minority had 5 features (4.9%). Significant differences in gender were observed in the prevalence rates of MetS (*p* < 0.001), MetS features (*p* < 0.001) and number of MetS features (*p* = 0.044). Women showed a significantly higher prevalence of “high WC” and “low HDL-C” components; all the other features were more prevalent in men. The prevalence of the number of MetS features also varied according to sex. The presence of one or less components was higher in men, and the presence of two to four components was higher in women. Regarding the geographical distribution of MetS and after adjustment for age and gender, significant differences were observed for the 18 districts of the Portugal mainland (Table 2); “Vila Real”, and “Leiria” districts had a higher prevalence of MetS; however, “Bragança” and “Beja” districts presented a lower prevalence. However, no differences were observed when we compared the syndrome prevalence according to NUTS2 statistical regions, which establish a north-south division of the country (Table 3).

**Table 1:** Metabolic syndrome age-adjusted prevalence and its individual features according to different proposed definitions

	*n*	Total [% (95%CI)]	Women [% (95%CI)]	Men [% (95%CI)]	*p*
MetS definition					
ATP III	3986	36.5 (34.3–38.6)	38.8 (36.2–41.4)	33.5 (30.8–36.2)	<0.001
IDF	3986	49.6 (47.5–51.7)	52.0 (49.5–54.5)	46.5 (43.9–49.1)	<0.001
HARM2009	3987	43.1 (41.0–45.3)	45.7 (43.2–48.3)	39.8 (37.2–42.4)	<0.001
MetS features ^a^					
Waist circumference	3977	51.0 (48.9–53.1)	66.2 (63.6–68.7)	35.7 (33.2–38.2)	<0.001
Glucose	3965	24.9 (23.0–26.8)	20.8 (18.8–22.8)	32.0 (29.2–34.8)	<0.001
Triglycerides	3980	29.4 (27.3–31.5)	24.7 (22.5–27.0)	37.2 (34.1–40.4)	<0.001
HDL cholesterol	3984	56.5 (54.4–58.6)	61.2 (58.6–63.7)	50.6 (47.9–53.3)	<0.001
Blood pressure	3984	64.3 (60.8–67.8)	60.6 (56.6–64.5)	69.7 (64.9–74.5)	<0.001
Number of MetS features ^a^					
0	530	8.9	8.4	9.7	
1	846	20.4	19.3	21.8	
2	998	26.2	25.9	26.5	
3	874	23.3	24.9	21.2	
4	518	16.3	16.6	15.7	
5	182	4.9	4.9	5.0	
Mean (95%CI)		2.29 (2.23–2.35)	2.32 (2.26–2.39)	2.24 (2.17–2.31)	0.044

MetS: metabolic syndrome.

The PORMETS study was conducted in mainland Portugal from February 2007 to July 2009.

^a^Metabolic syndrome features defined according to HARM2009.

**Table 2:** Metabolic syndrome prevalence (as defined by HARM2009) by Portuguese district

District	Prevalence (95%CI)	Prevalence ratio (95%CI)	Prevalence (95%CI)	Prevalence ratio (95%CI)
	Crude		Adjusted for age and sex	
Viana do Castelo	42.3 (35.8–48.8)	1.07 (0.91–1.23)	45.9 (39.6–52.1)	1.06 (0.92–1.20)
Braga	28.5 (22.7–34.2)	0.72 (0.59–0.86)	39.2 (32.1–46.2)	0.91 (0.75–1.07)
Vila Real	50.6 (44.2–57.0)	1.28 (1.12–1.44	55.1 (49.3–60.9)	1.27 (1.15–1.39)
Bragança	31.7 (25.8–37.6)	0.80 (0.66–0.95)	35.9 (29.7–42.1)	0.83 (0.70–0.97)
Porto	47.6 (41.1–54.1)	1.20 (1.04–1.37)	47.3 (41.4–53.2)	1.10 (0.97–1.22)
Aveiro	39.5 (33.2–45.8)	1.00 (0.85–1.16)	42.4 (36.2–48.7)	0.98 (0.85–1.11)
Viseu	29.4 (23.5–35.2)	0.74 (0.61–0.89)	38.4 (31.7–45.1)	0.89 (0.74–1.04)
Guarda	43.6 (37.2–49.9)	1.10 (0.95–1.26)	45.1 (39.1–51.1)	1.04 (0.91–1.17)
Coimbra	34.7 (28.7–40.7)	0.88 (0.74–1.03)	39.8 (33.4–46.1)	0.92 (0.78–1.06)
Leiria	48.0 (41.1–54.9)	1.21 (1.04–1.39)	50.7 (44.4–57.0)	1.17 (1.03.1.31)
Castelo Branco	44.0 (37.7–50.3)	1.11 (0.96–1.27)	44.7 (38.8–50.6)	1.04 (0.91–1.16)
Santarém	38.1 (31.9–44.3)	0.96 (0.82–1.12)	43.8 (37.5–50.1)	1.01 (0.88–1.15)
Lisbon	46.0 (39.7–52.3)	1.16 (1.01–1.32)	40.2 (34.7–45.7)	0.93 (0.81–1.05)
Portalegre	43.5 (36.0–50.9)	1.10 (0.92–1.28)	49.4 (42.5–56.2)	1.14 (0.97–1.30)
Évora	41.4 (35.2–47.7)	1.05 (0.90–1.20)	42.8 (37.0–48.6)	0.99 (0.86–1.12)
Setúbal	38.5 (26.6–50.3)	0.97 (0.70–1.26)	46.4 (34.9–58.0)	1.07 (0.80–1.33)
Beja	34.2 (28.1–40.4)	0.86 (0.72–1.02)	34.3 (28.3–40.2)	0.79 (0.67–0.92)
Faro	39.8 (33.9–45.8)	1.01 (0.86–1.16)	41.8 (36.1–47.5)	0.97 (0.84–1.09)

Prevalence considering a 53.2-year mean age and a 42.2% proportion of men in the sample.

Reference class for prevalence ratio estimation:” Deviation coding” – comparison of the individual districts with its global geometric mean.

The PORMETS study was conducted in mainland Portugal from February 2007 to July 2009.

**Table 3:** Metabolic syndrome prevalence (as defined by HARM2009) by Portuguese NUTS

NUTS II	Prevalence (95%CI)	Prevalence ratio (95%CI)	Prevalence (95%CI)	Prevalence ratio (95%CI)
	Crude		Adjusted for age and sex	
North	40.0 (37.2–42.8)	0.98 (0.90–1.07)	45.0 (41.9–48.1)	0.96 (0.89–1.04)
Center	39.6 (37.1–42.2)	1.00 (0.93–1.07)	43.4 (40.5–46.3)	1.06 (0.99–1.13)
Lisbon	41.7 (37.5–45.8)	0.99 (0.92–1.06)	41.8 (37.7–45.9)	1.02 (0.96–1.08)
Alentejo	39.4 (35.6–43.2)	1.04 (0.95–1.13)	40.8 (40.0–44.6)	0.98 (0.91–1.06)
Algarve	39.8 (33.9–45.8)	0.99 (0.87–1.12)	41.6 (35.9–47.3)	0.96 (0.88–1.03)

Prevalence considering a 53.2-year mean age and a 42.2% proportion of men in the sample.

Reference class for prevalence ratio estimation:” Deviation coding” – comparison of the individual NUTS II with its global geometric mean.

The PORMETS study was conducted in mainland Portugal from February 2007 to July 2009.

In Table 4, results from the comparison between participants with and without MetS are presented according to demographic, behavioural, anthropometric and analytical characteristics. As expected, participants with MetS had significantly higher mean values of Wt, BMI and WC. Regarding analytical characteristics, participants with MetS had significantly higher mean levels of glucose and triglycerides (*p* < 0.001). The insulin level and HOMA were also significantly higher in participants with MetS (*p* < 0.001). In addition, individuals with MetS syndrome significantly reported a higher prevalence of previously diagnosed type 2 diabetes (*p* < 0.001), myocardial infarction (*p* < 0.001) and stroke (*p* = 0.001). MetS was significantly more frequent in women and older subjects (*p* < 0.001). In addition, the syndrome was more common in housewives (*p* = 0.010), retired (*p* = 0.046) or unemployed (*p* = 0.024) participants. However, MetS was less frequent in smokers (*p* = 0.001) and in those that reported regular physical exercise (*p* < 0.001).

**Table 4:** Demographic, behavioural and analytical characteristics of the Metabolic Syndrome subjects

	With MetS	Without MetS	Prevalence ratio (95%CI) ^a^	*p*
Gender Men	656 (39.0)	1026 (61.0)	*	
Women	938 (40.7)	1367 (59.3)	1.15 (1.07–1.23)	< 0.001
Age [years, n (%)] 18–30	21 (5.0)	402 (95.0)	*	
31–40	87 (16.0)	458 (84.0)	3.20 (2.02–5.06)	< 0.001
41–50	222 (30.5)	506 (69.5)	6.09 (3.96–9.37)	< 0.001
51–60	391 (47.3)	435 (52.7)	9.52 (6.23–14.53)	< 0.001
61–70	467 (58.5)	331 (41.5)	11.91 (7.82–18.15)	< 0.001
> 70	406 (60.9)	261 (39.1)	12.40 (8.14–18.90)	<0.001
Education [years, n (%)] 0–4	1054 (53.2)	929 (46.8)	1.11 (0.97–1.28)	0.137
5–12	415 (27.1)	1116 (72.9)	1.03 (0.89–1.20)	0.678
> 12	125 (26.4)	348 (73.6)	*	
Marital status Single/divorced/widower	377 (34.6)	713 (65.4)	*	
Married	1211 (41.9)	1676 (58.1)	0.99 (0.92–1.08)	0.869
Occupation [n (%)] Student	2 (2.4)	81 (97.6)	0.54 (0.14–2.09)	0.372
Unemployed	72 (36.2)	127 (63.8)	1.26 (1.03–1.53)	0.024
Housewife	195 (48.4)	208 (51.6)	1.23 (1.05–1.43)	0.010
Retired	703 (58.4)	500 (41.6)	1.16 (1.00–1.35)	0.046
*Blue-collar*	251 (29.4)	602 (70.6)	1.03 (0.81–1.20)	0.684
*White-collar*	193 (24.1)	607 (75.9)	*	
Smoking status [n (%)] Smoker	137 (24.2)	429 (75.8)	0.85 (0.73–0.98)	0.027
Ex-smoker	245 (42.8)	327 (57.2)	1.00 (0.90–1.11)	0.977
Non-smoker	1198 (43.1)	1580 (56.9)	*	
Physical exercise [n (%)] No	1232 (42.9)	1638 (57.1)	1.18 (1.08–1.29)	<0.001
Yes	338 (31.9)	722 (68.1)	*	
Residence [n (%)] Non-urban	835 (43.4)	1087 (56.6)	1.13 (1.05–1.20)	0.001
Urban	759 (36.8)	1306 (63.2)	*	
Weight [kg, Mean (SE)]	78.5 (13.6)	68.1 (12.3)	1.00 (1.00–1.00)	<0.001
Body mass index [kg/m^2^, Mean (SE)]	30.0 (4.4)	25.6 (4.0)	1.09 (1.08–1.10)	<0.001
Waist circumference [cm, Mean (SE)]	101.4 (10.1)	88.2 (11.0)	1.00 (1.00–1.00)	<0.001
Hip circumference [cm, Mean (SE)]	108.2 (9.4)	100.5 (8.5)	1.00 (1.00–1.00)	<0.001
Total cholesterol [mg/dL, Mean (SE)]	211.6 (43.0)	207.1 (41.1)	1.04 (0.96–1.13)	0.305
HDL cholesterol [mg/dL, Mean (SE)]	43.2 (11.0)	51.0 (12.5)	0.81 (0.65–1.06)	0.067
Triglycerides [mg/dL, Mean (SE)]	159.0 (86.4)	99.8 (46.3)	1.28 (1.24–1.32)	<0.001
Glucose [mg/dL, Mean (SE)]	105.5 (35.7)	83.1 (16.1)	1.11 (1.07–1.15)	<0.001
Insulin [μU/ml, Median (P25 -P75)]	9.99 (6.80–14.30)	6.50 (4.40–9.50)	1.03 (1.03–1.04)	<0.001
hs-CRP [mg/L, Median (P25 -P75)]	0.22 (0.10–0.47)	0.13 (0.06–0.29)	1.00 (1.00–1.00)	0.107
HOMA –IR [Median (P25 -P75)]	2.43 (1.58–3.72)	1.32 (0.86–1.96)	1.09 (1.07–1.10)	<0.001
Diabetes [n (%)]	451 (76.8)	136 (23.2)	1.65 (1.55–1.75)	< 0.001
Previous myocardial infarction [n (%)]	115 (69.3)	51 (30.7)	1.26 (1.14–1.40)	<0.001
Previous stroke [n (%)]	138 (63.3)	80 (36.7)	1.19 (1.07–1.31)	0.001

MetS: metabolic syndrome (as defined by HARM2009).

The PORMETS study was conducted in mainland Portugal from February 2007 to July 2009.

^a^Prevalence ratio adjusted for sex and age.

*Reference class.

When we compared the prevalence of the syndrome, according to the classification of the health care centre location in an urban/non-urban area, we found that MetS was significantly (*p* = 0.001) more prevalent in non-urban subjects after adjustment for sex and age (PR:1.13;95%CI of 1.05 to 1.205). Non-urban residents were discretely older (mean age of 53.5 versus 52.9 years; *p* = 0.229), presenting a slightly higher proportion of men (44.0 versus 40.8%; *p* = 0.040) and had a lower education level (mean of 6.0 versus 7.5 schooling years; *p* < 0.001). After additional adjustment for the education level, non-urban residents maintained a higher PR for MetS (1.17; 95%CI of 1.06 to 1.29).

## Discussion

The crude prevalence of MetS in our population-based survey varied according to the definition used. The HARM2009 definition, supported by several major organizations [7], is the most consensual. The ATP III and IDF definitions have been widely used and may be useful for comparisons of the prevalence of MetS between studies. The prevalence was lowest according to the ATP III definition (32.7%), followed by the HARM2009 definition (40%), and was highest when using the IDF definition (45.9%). These differences were confirmed by previous studies [13]. The cut-off points of the WC component in the IDF setting were lower, and the cut-off points of glycaemia in the ATP III definition were higher than those using the HARM2009 definition.

The high prevalence of MetS is supported by previous Portuguese studies [13–15]. A study in the city of Porto, conducted during 1999–2003, and including a younger population (mean age of 52.5 years) recruited with a different methodology, estimated lower MetS crude prevalence rates (24.0%, 41.9% and 27.6% according to the ATP III, IDF and HARM2009 definitions, respectively) [13]. Another national study [15], carried out between 2006 and 2007, with different selection criteria and including an older population (mean age of 58.1 years), observed crude MetS prevalence rates of 28.4%, 65.5% and 69.4%, respectively, discretely higher than those in the present study. The PREVADIAB study conducted from 2008 to 2009 showed a crude prevalence of 41.5% by IDF criteria [14]. Nevertheless, the national prevalence of MetS is higher than in some European countries and the USA [8].

The high prevalence of MetS in the Portuguese population may partly be explained by the decreasing adherence of the Portuguese population to the Mediterranean diet [18] and high prevalence of sedentary lifestyles, namely in older adults [19], hypertension [11], obesity [9] and type 2 diabetes [12].

Hypertension is highly prevalent among Portuguese adults [11] but, as observed in other countries [20], a decreasing trend in the last decade was observed [21].

The prevalence of obesity in Portugal [9] and worldwide [22] has risen to epidemic/pandemic proportions. A systematic review on the prevalence of overweight and obesity in Portugal (1995–2005) showed an increase in the overweight prevalence by 3.2% and 3.5% and in the obesity prevalence by 7.4% and 1.3% among women and men, respectively [10]. In addition, diabetes is rising globally [23], and Portugal has one of the highest prevalence rates [12] compared with other European countries [24].

Our study showed a higher prevalence of the “low HDL-C” component than that reported in previous MetS prevalence Portuguese studies [13, 25]. Nevertheless, a recent systematic review that summarizes the evidence from Portuguese studies [26] showed mean HDL-C values discretely higher than ours, for both sexes.

According to our data, the prevalence of the “high triglycerides” component was discretely lower than that in previous Portuguese studies [13, 25]. However, a systematic review [26] that quantified the distribution of lipid fractions found mean triglycerides levels of 150 mg/dL and 111 mg/dL in men and women respectively, similar to our results.

The presence of MetS increases the risk of developing CVD [1] and type 2 diabetes [2]. This study showed that subjects with MetS significantly reported a higher prevalence of previously diagnosed type 2 diabetes, myocardial infarction and stroke, suggesting the association of MetS with CVD and diabetes. However, in Portugal, although the prevalence of MetS is high, the age-adjusted mortality rates from CVD are low, namely when compared with Western Europe countries [27], and have been decreasing in the last decade [28].

As expected, higher insulin levels and HOMA scores were found in the individuals with MetS. These results are supported by a previous study in the city of Porto [29], stressing the contribution of insulin resistance to the atherogenic profile of the syndrome [30].

According to our data, sedentary behaviour was associated with a higher prevalence of MetS. These results are supported by a recent meta-analysis [31].

Regarding this health problem in our societies, we must consider not only biological but also socio-demographic and psychosocial conditions. Some groups, such as the elderly and the most socioeconomically disadvantaged have a higher risk of MetS [32]. Our data showed an association of MetS with being a housewife, retired or unemployed, corroborating findings from another Portuguese study [33] and from other developed countries [34, 35].

Some differences were found between districts but not by regions of the Portugal mainland (NUTS II). In addition, differences between urban and non-urban populations in the prevalence of the MetS were observed, with this condition being more frequent in non-urban areas. The data from the 1999–2006 National Health and Nutrition Examination Survey also showed that non-urban dwelling was associated with a higher prevalence of MetS among adults in the United States [36]. This disparity, which was also found in other countries [37], may be explained by demographic and socio-economic factors [38]. However, these factors were addressed in this study and the differences persisted; other explanations must be proposed.

Our study did not address the participants’ eating patterns. We cannot exclude the contribution of the food pattern to the differences found in some regions and non-urban areas.

Given the increasing global burden of noncommunicable diseases such as diabetes and CVD, which is driven by forces that include ageing and unhealthy lifestyles, the estimation of the modifiable behavioural, metabolic and physiological risk factors is essential. This study, when documenting a high prevalence of metabolic syndrome in Portugal, with all the risks involved, calls attention to the need for an early diagnosis and therapeutic intervention. The study also draws attention to at-risk groups that deserve special attention. These groups include those living in non-urban areas or districts with a higher prevalence of MetS. The elderly, women, the most disadvantaged socio-economic categories and individuals with overweight or sedentary lifestyles should also be considered.

Our study includes some strengths, namely the sample size and selection of participants by districts and consideration of urban and non-urban residence. Although our study intends to be representative of the adult Portuguese population, we did not consider individuals not enrolled at health centres belonging to the national health system; however, all citizens have universal access to the national health system. Another limitation of our study was the lack of control of the participants’ eating patterns.

## Conclusions

This study showed that MetS is highly prevalent in the Portuguese adult population. A high prevalence of hypertension, obesity and diabetes in Portugal, may contribute to these numbers.

Ageing [39] and the trend towards increasing obesity in Portugal [10] are expected to contribute to a future increase in MetS prevalence. Differences in the prevalence of this syndrome were observed by district. In addition, this condition was more frequent in non-urban areas. These results may be useful in selecting priority sites for future national intervention.

Our study provides valuable baseline information for the development of future interventions in Portugal and to assess the trends in the evolution of the MetS and associated risk factors.

## Abbreviations

MetS: Metabolic syndrome
CVD: Cardiovascular disease
HDL-C: High-density lipoprotein cholesterol
IDF: International Diabetes Federation
HARM2009: Harmonizing criteria
Wt: Weight
BMI: Body mass index
WC: Waist circumference
HC: Hip circumference
hs-CRP: High sensitivity C-reactive protein
HOMA: Homeostatic model assessment
ATP III: Adult treatment panel III
SD: Standard deviation; PR: Prevalence ratio
